# Establishment to measure oxycodone in plasma with liquid chromatography–tandem mass spectrometry

**DOI:** 10.1002/npr2.12268

**Published:** 2022-06-11

**Authors:** Suguru Ito, Masato Mori, Momoka Matsuo, Rio Yamasaki, Yasuhisa Oida, Midori Soda, Kiyoyuki Kitaichi

**Affiliations:** ^1^ Laboratory of Pharmaceutics, Department of Biomedical Pharmaceutics Gifu Pharmaceutical University Gifu Japan

**Keywords:** liquid chromatography, opioids, oxycodone, plasma, solid‐phase extraction

## Abstract

Oxycodone (OXY) is classified as a “strong opioid” in the World Health Organization system of cancer pain treatment. However, OXY also causes severe adverse reactions, such as respiratory depression. Thus, in order to adjust the dosage of OXY for well‐managed pain relief with less toxicity, we tried establishing and validating a system for measuring plasma concentrations of OXY using liquid chromatography–tandem mass spectrometry (LC–MS/MS). Human pooled plasma samples containing OXY diluted with 0.1% formic acid solution and internal standard (papaverine) were used for solid‐phase extraction. The eluents were injected into LC–MS/MS with Unison UK‐Silica column (100 × 2 mm, 3 μm, Imtakt). Mobile phase was a mixture of 1 mM ammonium formate solution and acetonitrile containing 0.1% formic acid (50:50). OXY in plasma could be measurable with good linearity in a concentration range of 2–100 ng/ml by using 100 μl of plasma within 4 min. Relative standard deviations of all validation results were within ±15%. These results suggest that our established method using LC–MS/MS to measure OXY in plasma would be useful to adjust the dosage of OXY in order to obtain its efficacy and to avoid its adverse reactions.

## 1 INTRODUCTION

Opioids are widely used to treat severe pain caused by cancer and other diseases.^1^, ^2^ Oxycodone (OXY), a semi‐synthetic opioid derived from thebaine, is a narcotic analgesic, μ‐opioid receptor agonist.^3^ In the World Health Organization (WHO) guidelines for treating cancer pain, OXY is classified as a “strong opioid” along with morphine and fentanyl.^4^ Global OXY use has increased significantly over the past two decades^5^ since OXY shows higher bioavailability, and it is easier to use in patients with renal dysfunction than morphine.^6^, ^7^, ^8^, ^9^ In October 2020, an indication for treating non‐cancer chronic pain was added to OxyContin TR Tablets (Shionogi & Co., Ltd., Osaka) sold in Japan, an oral extended‐release formulation containing OXY. Actually, Kawamata et al reported that the efficacy and safety of this drug in patients with non‐cancer chronic pain is equivalent to that of existing OXY drugs.^10^

OXY overdose has severe adverse reactions, such as coma, convulsions, bradycardia, respiratory depression, and death.^11^, ^12^ Also, adverse reactions, such as respiratory depression,^13^ and QT prolongation,^14^ correlate with OXY dosage. However, the range of plasma OXY concentrations for pain relief varies between patients due to various backgrounds (age, cancer progression, history of opioid therapy, and pain sensitivity) and interactions with concomitant medications.^15^, ^16^

Thus, in order to adjust the appropriate dose of OXY in each patient, we attempted to establish a simple and quick method to measure plasma concentrations of OXY by liquid chromatography–mass spectrometry (LC–MS/MS).

## 2 METHODS

### 2.1 Chemicals and reagents

OXY injection (10 mg/ml) was purchased from Shionogi & Co. (Osaka, Japan). Papaverine (PAV), used as the internal standard (IS), was purchased from Nichi‐Iko Corporation (Toyama, Japan). Formic acid, ammonium formate, acetonitrile (MeCN), and methanol (MeOH) were purchased from Fujifilm Wako Pure Chemicals Co. (Osaka, Japan). Diluted water for LC–MS was purchased from Hikari Seiyaku Co. (Osaka, Japan), and human pooled plasma was purchased from Cosmo Bio Co. (Tokyo, Japan).

### 2.2 Liquid chromatography

The liquid chromatography system (Shimadzu Co., Kyoto, Japan) comprised a binary pump (LC‐20 AD), an autosampler (SIL‐30 AC), and column oven (CTO‐10A) with Unison UK‐Silica column (2 × 100 mm, particle size 3 μm, Imtakt, Kyoto, Japan) maintained at 40°C. The mobile phase comprised 1 mM ammonium formate in water and MeCN containing 0.1% formic acid (50/50, v/v). The flow rate was set to 0.13 ml/min. The injection volume was 2 μl, and the run time was 4 min.

### 2.3 Mass spectrometry

The determination of OXY and IS was conducted using an LC–MS‐8045 triple quadrupole mass spectrometer (Shimadzu Co., Kyoto, Japan). The positive mode of electrospray ionization (ESI) was selected as the ionization method. Additionally, the MS parameters were optimized with auto‐optimized software by infusing standard solutions of OXY and IS (100 ng/ml). Optimal parameters were as follows: nebulizer gas (nitrogen) 3.0 L/min; desolvation line temperature 250°C; heat‐block temperature 400°C; drying gas (nitrogen) 10.0 L/min. Q1 Pre vias, collision energy, and Q3 pre vias were 16, 19, and 20 V for OXY, and 17, 30, and 21 V for IS, respectively. The transitions (*m*/*z*; precursor ion → product ion) for multiple reaction monitoring were 316.0 → 298.2 for OXY and 340.4 → 324.1 for IS. Data acquisition and integration were controlled using Lab Solutions LCMS software (Shimadzu Co.).

### 2.4 Preparation of working solution and quality control sample

First, the stock solution of PAV was made by dissolving it in distilled water at 10 μg/ml and was stored at −30°C until use. Then, OXY injection (10 mg/ml) in an ampoule was used as a stock solution for making the standard solution when needed. The working solutions of OXY and IS were prepared by diluting their stock solutions with distilled water immediately before use. In the case of standard samples for the calibration curve, OXY working solution at designated concentrations was added to human plasma. IS solution was prepared for 50 ng/ml by diluting the IS stock solution. Finally, quality control (QC) samples spiked with OXY were prepared for method validation, and final concentrations of low‐quality control (LQC), medium‐quality control (MQC), and high‐quality control (HQC) samples were 4, 20, and 80 ng/L, respectively.

### 2.5 Sample extraction

Load samples were prepared by mixing 100 μl of plasma, 50 μl of IS solution, 300 μl of 0.1% formic acid, and 50 μl of OXY diluent. In the case of QC samples, 100 μl of plasma spiked with OXY, 50 μl of IS solution, and 350 μl of 0.1% formic acid were mixed. After mixing, OXY extraction was performed using the solid‐phase extraction (SPE) method. Then, loading samples (500 μl) were loaded on Oasis hydrophilic–lipophilic balance Extraction Cartridges 1 cc, 10 mg (Waters, Milford, USA) conditioned by 1.0 ml MeOH and 1.0 ml of 0.1% formic acid solution. After washing the SPE cartridge with 1.0 ml of 5% MeOH, OXY, and IS were eluted with 500 μl of a mixture of MeCN and MeOH (MeCN:MeOH = 9:1). The eluate was completely dried under a gentle nitrogen stream, and the residue was redissolved in 200 μl of 50% MeCN solution. It was then filtered using Millex‐LG (0.20 μm, Merck, Darmstadt) for LC–MS/MS measurements.

### 2.6 Calibration standards

The calibration curve was prepared at n = 2 for each sample to obtain 2, 4, 20, 40, 80, and 100 ng/ml plasma concentrations. The regression line was obtained by linear regression. Using the regression equation, the plasma OXY concentration was calculated from each sample’s OXY/IS ratio.

### 2.7 Method validation

The method was validated according to the Food Drug Administration guidance^17^ for biological method validation. The raw data are available in the Supporting Information data files.

### 2.8 Specificity and selectivity

Selectivity and specificity were evaluated by comparing the chromatograms obtained by measuring blank plasma (without OXY and IS) and plasma containing OXY and IS (at plasma concentrations of 20 ng/ml and 25 ng/ml, respectively). This evaluation was performed to confirm that no plasma‐derived peaks were detected in the retention times of OXY and IS.

### 2.9 Intra‐ and inter‐day precision and accuracy

Intra‐assay precision and accuracy were evaluated by repeating analyses of QC samples in five independent runs at three OXY concentrations (LQC, MQC, and HQC) in one day. Inter‐assay precision and accuracy were evaluated by analyzing three QC samples on five different days. Accuracy was considered acceptable when the relative error was within ±15%, and precision was considered acceptable at a relative standard deviation (RSD) of less than 15% except at lower limit of quantification should not deviate by more than 20%.

### 2.10 Recovery

Recovery rates were evaluated by comparing the peak area ratio when the sample was pretreated with OXY standard solution in human plasma and QC samples. Also, the peak area ratio when the standard solution of OXY was spiked after sample processed of human plasma.

Recovery (%) = (response of sample #1)/(response of sample #2) × 100.

Sample #1: OXY and IS were extracted from blank plasma using SPE; Sample #2: OXY and IS were added to eluted samples and were adjusted to theoretical concentrations.

### 2.11 Stability

The stability of OXY was determined in LQC (4 ng/ml), MQC (20 ng/ml), and HQC (80 ng/ml) samples. The post‐preparative stability of OXY in injection solvent was evaluated by injecting prepared samples after storage at 8°C in the autosampler for 8 h from the first cycle injection. At the initial cycle, peak area ratios of OXY and IS were used as standards to determine 8 h stability at corresponding concentrations. The bench‐top stability of OXY in human plasma was evaluated at room temperature for 18 h. The freeze–thaw stability of OXY in human plasma following three freeze–thaw cycles was assessed by analyzing QC samples after the third round of thawing. In these experiments, samples were thawed at room temperature, frozen, and stored at −30°C until the next thaw. The short‐term stability of OXY in human plasma was assessed by analyzing QC samples after storage at −30°C for 2 weeks. The long‐term stability of OXY in human plasma was assessed by analyzing QC samples after storage at −30°C for 6 months. The stability of stock OXY in diluting solvent was assessed by analyzing OXY stock solution after storage at −30°C for 6 months.

### 2.12 Carryover

Carryover peak was assessed by checking peaks in OXY and IS peak regions of blank sample (no OXY or IS) injections after HQC (80 ng/ml) sample injection.

### 2.13 Matrix effects

The matrix effect of human plasma components was evaluated from the ratio of OXY and IS ionization reactions between human blank plasma samples and samples of standards (4, 20, and 80 ng/ml) diluted in purified water.

Matrix effect = (response of sample #1)/(response of sample #3).

Sample #1: OXY and IS were extracted from blank plasma using SPE; Sample #3: OXY and IS were extracted from distilled water using SPE.

## 3 RESULTS

### 3.1 Mass spectrometry and liquid chromatography condition

The mobile phase and column used in this method were selected based on evaluating the shape of the obtained peaks and reproducibility. ESI conditions were optimized for OXY and IS, and Q1 pre‐rod bias, collision energy, and Q3 pre‐rod bias were determined in the positive mode. Furthermore, the product ions produced from the precursor ion of OXY (*m*/*z* = 316.0) were, in order of intensity, *m*/*z* 298.2, 256.1, and 241.2. However, the product ions at *m*/*z* 298 and 241 were also used to measure OXY reported in previous studies.^18^, ^19^, ^20^, ^21^ In this study, *m*/*z* 298.2 was selected because of quantification’s peak shape and stability. In the same way, *m*/*z* 324.1 was detected as the main fragment ion in PAV (IS). Therefore, *m*/*z* 324.1 was selected as the product ion in this study.

### 3.2 Specificity, selectivity, and linearity

**FIGURE 1:**
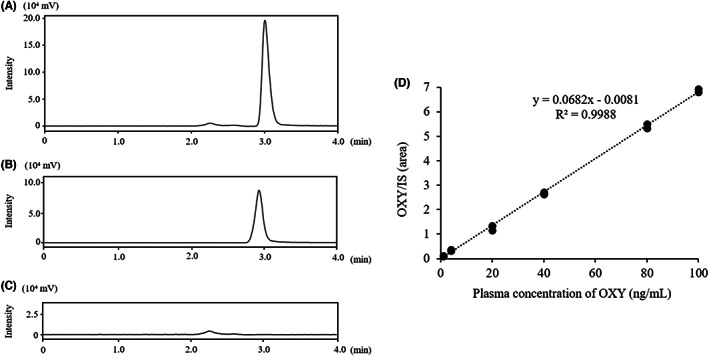
Typical chromatograms of OXY and IS in plasma. (A) 20 ng/ml of OXY; (B) 50 ng/ml of PAV (IS); (C) Blank plasma (OXY‐ and IS‐free sample); (D) Standard curve (n = 2)

Typical chromatograms of OXY and IS are shown in Figure 1A,B, and the results of blank plasma samples without OXY and IS are shown in Figure 1C. No background peaks interfering with the OXY and IS peaks were observed. Also, OXY showed good linearity with an *R* ^2^ value > 0.99 in the range of 2–100 ng/ml plasma concentration (Figure 1D).

### 3.3 Intra‐ and inter‐day precision and accuracy

Intra‐ and inter‐day precision and accuracy in human plasma samples are shown in Table 1. The results were within the acceptable range (<15%) in all cases, and good reproducibility was confirmed.

**TABLE 1:** Intra‐day and inter‐day precision and accuracy of OXY determinations

OXY spiked conc. (ng/ml)	Found C (ng/ml)	Intra‐day (n = 5)		Inter‐day (n = 5)	
		RSD (%)	RE (%)	RSD (%)	RE (%)
80	79.5 ± 3.2	7.0	−2.7	4.1	−0.7
20	20.1 ± 0.6	4.2	1.7	2.8	0.4
4.0	4.0 ± 0.3	2.9	9.7	7.6	0.04
2.0	1.9 ± 0.08	4.3	−6.8	3.9	−3.9

*Note*: Found C is the measured concentration of OXY in spiked plasma. Abbreviations: RSD: relative standard deviation; RE: relative error. Data of Found C represent mean ± SD.

### 3.4 Recovery and matrix effects

Recovery and matrix effects in human plasma samples are shown in Table 2. Recovery rates of OXY were reproducible at all concentrations with standard deviations less than 5%. Matrix effects in this method did not affect the measurement of the target.

**TABLE 2:** Recovery and matrix effect of OXY determinations

	OXY (ng/ml)		
	80	20	4
Recovery (%, n = 5)	102.6 ± 3.6	111.1 ± 2.6	102.0 ± 4.3
Matrix effects (%, n = 3)	104.0 ± 0.5	107.6 ± 3.1	101.9 ± 3.6

*Note*: Mean ± SD.

### 3.5 Stability

The results of various stability studies are shown in Table 3. All stability tests were met the acceptable criteria (<15%). Stock solution was also stable up to 6 months at −30°C (supporting information).

**TABLE 3:** Stability of OXY in human plasma under different storage conditions

Storage condition	OXY (%RSD)		
	HQC	MQC	LQC
	80 ng/ml	20 ng/ml	4 ng/ml
Post‐preparative stability 8 h in autosampler at 8°C	0.3	−0.2	0.3
Bench‐top stability 18 h at room temperature	−7.8	−4.6	−8.1
Freeze–thaw stability^a^	−1.3	1.0	−1.0
Short‐term stability 2 weeks at −30°C	−0.6	2.0	2.8
Long‐term stability 6 months at −30°C	−14.9	−11.2	−0.2

*Note*: Each data represents as %RSD (N = 3).

^a^ OXY was measured after three freeze (−30°C) ‐thaw (room temperature) cycles.

### 3.6 Carryover

No significant peaks were detected from blank samples after injections of HQC samples. Therefore, we can conclude that this measurement has no carryover (data not shown).

## 4 DISCUSSION

OXY is a drug to treat cancer and non‐cancer chronic pain.^3^, ^22^, ^23^ However, considering its adverse reactions at overdose^11^, ^12^, ^13^, ^14^, ^24^ and the variation of the dose of OXY for pain relief between patients,^15^, ^16^ the monitoring of plasma concentration of OXY would be needed to adjust its dosage. Thus, we tried to establish and to validate the method for measuring the plasma concentration of OXY.

Several methods have already been established to measure plasma concentrations of OXY using LC–MS/MS. There are several reports to simultaneously quantify the metabolites of OXY.^25^, ^26^ OXY is mainly metabolized to noroxycodone, the major metabolite, by N‐demethylation by CYP3A4/5 and to oxymorphone by O‐demethylation by CYP2D6. Furthermore, noroxycodone and oxymorphone are further metabolized to noroxymorphone by CYPs and glucuronosylated by UDP‐glucuronosyltransferase and then excreted from the body. A previous report has demonstrated that the maximum plasma concentration of noroxycodone, oxymorphone, and noroxymorphone relative to the maximum blood concentration (Cmax) (mol/L) of OXY, were 72%, 3%, and 23%, respectively.^27^ Among these metabolites, noroxycodone and noroxymorphone have no analgesic activity. Although oxymorphone has stronger analgesic activity than OXY, its production is low.^28^ Thus, it is crucial to measure OXY rapidly to investigate the relationship between plasma OXY concentration and its analgesic activity, and this assay is suitable for achieving this purpose.

Our measurement system is highly quantitative in the range of 2–100 ng/ml. The intra‐ and inter‐day validation experiments showed good reproducibility with RSD values at <8%. The stability of OXY was also confirmed in spiked plasma. Our method detected both OXY and IS at about 3.0 min. A total run time is 4 min, which is approximately 1 min shorter than that in previous reports.^25^, ^26^ Moreover, the volume of eluent (500 μl) during SPE in our method was also less than those (2000–3000 μl) in previous reports,^26^, ^27^ which could contribute to reduce the time required for drying the eluents. Additionally, the amount of plasma used in our method is 100 μl, which is relatively smaller than that in previous reports (500–1000 μl)^26^, ^29^ except one^21^ although the range of standard curve is almost similar to that in these reports.^21^, ^26^, ^29^ Furthermore, the time spent in sample preparation was approximately 40 min. Among the previous reports investigated, the quality of our established method is mostly similar to the best method using centrifugal precipitation plate and UPLC condition (25 min preparation, 4.5 min measurement, 100 μl plasma needed, almost similar range of standard curve).^21^

The calibration curve range of this method is 2–100 ng/ml. Target plasma concentrations have not been established for OXY in pain treatment because of the wide variation in plasma concentration profiles among individuals. However, according to the pharmacokinetic study section of the package insert for OxyContin TR,^23^ an extended‐release formulation of oxycodone hydrochloride, the maximum plasma concentrations of OXY following a single administration of OxyContin TR (10 mg and 40 mg) on an empty stomach were 9.81 ± 2.74 ng/ml and 40.2 ± 10.8 ng/ml, respectively. Furthermore, according to a previous report, a comparison of the NRS (Numeric Rating Scale) and plasma concentration of OXY during postoperative pain management in patients with breast cancer showed that the plasma concentration of OXY was 17.8 ± 13.5 ng/ml in NRS 0–3, 31.6 ± 20.9 ng/ml in NRS 4–6, and 50.7 ± 24.3 ng/ml in NRS 7–10.^29^ Based on this information, the calibration curve of OXY in our method covered these ranges.

The IS method is widely used for LC–MS/MS measurements, in which a compound with a different exact mass from that of the substance to be measured (IS) is added to the sample. Furthermore, the substance to be measured is quantified based on the peak area ratio between the substance to be measured and the IS. In the measurement of plasma concentrations of OXY, there have been several reports of measurements using deuterated compounds such as oxycodone‐d3 as an IS because of its structural similarity to the target substance.^30^, ^31^ However, PAV, which was used in this study, is an inexpensive and readily available drug and is not readily absorbed along with OXY. Additionally, a method for measuring the plasma concentration of fentanyl using PAV as an IS for the same reason has been reported previously.^32^ Therefore, a PAV method as an IS is useful.

In conclusion, we successfully developed a rapid and simple method for measuring OXY concentrations in plasma using relatively small amount of plasma. Relatively rapid preparation of samples (approximately 40 min), quick measurement (4 min) with a relatively wide range (2–100 ng/ml) of the standard curve allow us to detect its varied plasma concentration from the therapeutic range to toxic range.^11^, ^12^, ^13^, ^14^, ^15^, ^16^ Thus, in the clinical practice, our improved method might contribute to quicker adjustment of the dose of OXY for well‐managed pain relief with less toxicity in each patient.

## AUTHOR CONTRIBUTIONS

Kiyoyuki Kitaichi, Yasuhisa Oida, and Midori Soda participated in research design. Suguru Ito, Masato Mori, Momoka Matsuo, and Rio Yamasaki performed experiments and analyzed data. Suguru Ito drafted the manuscript, which was revised and approved by all of the authors.

## CONFLICT OF INTEREST

The authors declare no conflict of interest.

## APPROVAL OF THE RESEARCH PROTOCOL BY AN INSTITUTIONAL REVIEWER BOARD

N/A.

## INFORMED CONSENT

N/A.

## REGISTRY AND THE REGISTRATION NO. OF THE STUDY/TRIAL

N/A.

## ANIMAL STUDIES

N/A.

## Supporting information


Supinfo

## Data Availability

The data that support the finding of this study are available within the article and its supplementary materials.
